# Biofilm model on mice skin wounds

**DOI:** 10.1590/acb370306

**Published:** 2022-06-01

**Authors:** Eline Lima Borges, Gilmara Lopes Amorim, Marina Barcelos de Miranda, Flaviano dos Santos Martins, Antônio Carlos Martins Guedes, Kinulpe Honorato Sampaio, Josimare Aparecida Otoni Spira, Lucíola da Silva Barcelos

**Affiliations:** 1 PhD. Universidade Federal de Minas Gerais – School of Nursing – Department of Basic Nursing – Belo Horizonte (MG), Brazil.; 2 MSc. Universidade Federal de Minas Gerais – School of Nursing – Postgraduate Program – Belo Horizonte (MG), Brazil.; 3 MSc. Universidade Federal de Minas Gerais – Institute of Biological Sciences – Department of Physiology and Biophysics – Belo Horizonte (MG), Brazil.; 4 PhD. Universidade Federal de Minas Gerais – Institute of Biological Sciences – Department of Microbiology – Belo Horizonte (MG), Brazil.; 5 PhD. Universidade Federal de Minas Gerais – School of Medicine – Medical Clinic Department – Belo Horizonte (MG), Brazil.; 6 PhD. Universidade Federal dos Vales Jequitinhonha e Mucuri – Diamantina Department of Medicine – Diamantina (MG), Brazil.; 7 MSc. Universidade Federal de Minas Gerais – School of Nursing – Department of Basic Nursing – Belo Horizonte (MG), Brazil.; 8 PhD. Universidade Federal de Minas Gerais – Institute of Biological Sciences – Department of Physiology and Biophysics – Belo Horizonte (MG), Brazil.

**Keywords:** Biofilms, Pseudomonas Infections, Wound Healing, Models, Animal

## Abstract

**Purpose:**

To evaluate a biofilm model of *Pseudomonas aeruginosa* in excisional cutaneous wound in mice.

**Methods:**

Preclinical, translational study conducted with 64 C57BL/6 mice randomly assigned to control and intervention groups. Evaluation was on days D0, D3, D5, D7 and D10 of wound making. The profile of biofilm formation and induction was evaluated using wound closure kinetics, quantitative culture, and evaluation of wounds using transmission electron microscopy (TEM). Clinical evaluation was performed by liver tissue culture, weight variation, and quantification of leukocytes in peripheral blood. Analyses were performed with GraphPad Prism software.

**Results:**

Bacterial load for induction of infection with *P. aeruginosa* and survival of animals was 10^4^ UFC·mL^-1^. In D5 (p < 0.0001) and D7 (p < 0.01), animals in the intervention group showed a delay in the healing process and had their wounds covered by necrotic tissue until D10. Statistical differences were observed in wound cultures and weight at D5 and D7 (p < 0.01). Liver cultures and leukocyte quantification showed no statistical differences. No bacteria in planktonic or biofilm form were identified by TEM.

**Conclusions:**

The findings raise questions about the understanding of the ease of formation and high occurrence of biofilm in chronic wounds.

## Introduction

Chronic skin wounds colonized or infected with bacterial biofilms are considered a public health problem worldwide due to the resistance of bacteria to host defense mechanisms and antimicrobial agents^1^^,^ 2. A meta-analysis study identified that the prevalence of bacterial biofilms in human chronic wounds is 78.2%^3^.

Biofilms can be found on the surface or in deeper granulation tissues, suspended in the exudate, adhered to necrotic tissue, or also in diverse dressings, widely used in the treatment of skin wounds^4^. Advances in understanding biofilm formation and development have not resulted in effective proposals of its clinical management^5^^,^ 6.

There is controversy about the conditions for clinical identification of biofilm, considering its microscopic nature. Inspection is a controversial point^5^. In care practice, some uncertain and imprecise criteria for identifying biofilm with the naked eye have been adopted. In such cases, the clinical sign considered is the presence of a shiny, translucent, viscous film on the wound surface that does not heal^7^^-^ 9. The presence of yellowish, loose, fluid, gelatinous material that rebuilds quickly after its removal is considered the signal for the presence of biofilm, in contrast to the sphacelus-like necrotic tissue, more adhered to the wound bed, which takes longer to recompose^10^^,^ 11.

Experimental *in vitro* and *in vivo* studies are important tools for evaluating and producing knowledge about biofilm. The use of *in-vitro* systems has allowed significant advances in understanding how antibiofilm agents work and serve as a premise for conducting more robust *in-vivo* clinical studies, such as preclinical trials and controlled clinical trials.

The results of studies published to date have not been sufficient to elucidate gaps in biofilm management and management in clinical practice. From this perspective, *in-vivo* (experimental) studies have been recommended as an alternative for questions from bench research to clinical research, providing a powerful basis to aid clinical decision making^12^.

The choice for the skin wound model with *in-vivo* biofilm became a cost-effective proposition when compared to the cost dimension of a clinical trial. Some researchers^13^^,^ 14 point out this experimental model as suitable and accurate for identifying the morphological structure of biofilm and for studying its behavior in skin wounds. It is inferred that the use of the experimental model in mice has as main advantages the possibility of inducing and following the formation of biofilm in an area predetermined by the researcher, greater control of the study variables and conditions of reproducibility of the wound model^15^. Considering this perspective, the study in an animal model becomes an important tool for understanding biofilm formation, its identification, and influence on tissue repair.

The creation of a feasible wound biofilm model is essential for a better understanding of the mechanisms involved in the processes of inflammation, angiogenesis, and tissue repair. It is believed that the biofilm model will subsidize the development of alternative studies to evaluate the effects of antimicrobial agents on biofilms, comparative studies to evaluate interventions that result in the reduction and elimination of biofilms. The results of these studies may yield improvements for the clinical management and management of biofilm in chronic wounds in clinical practice.

In order to advance this area of knowledge, this research aimed to evaluate a biofilm model of *Pseudomonas aeruginosa* in excisional cutaneous wound in mice.

## Methods

### Ethical considerations

The research was approved by the Ethics Committee on Animal Use of Universidade Federal de Minas Gerais (UFMG), under protocol number 87/2015. Upon completion of the wound biopsies and tissue collection for the proposed evaluations, the animals were euthanized according to the recommendations of the Ethics Committee on Animal Use of the UFMG and National Council for the Control of Animal Experimentation Normative Resolution No. 37, 2018.

### Design and scenario

This is a preclinical study, translational in nature. The experimental procedures were carried out in the Angiogenesis and Stem Cell Laboratory of the Department of Physiology and Biophysics and in the Laboratory of Biotherapeutic Agents of the Department of Microbiology, both at UFMG. The biofilm evaluation was performed at the Microscopy Center of UFMG.

### Determination of bacterial load

The bacterium used was *P. aeruginosa American Type Culture Collection* (ATCC) 25853 (ATCC 25853), sample (aliquot) provided by the Laboratory of Biotherapeutic Agents, Department of Microbiology, UFMG.

The sample of *P. aeruginosa* was propagated in 5 mL of brain and heart infusion (BHI) broth medium (manufacturer Acumedia) and grown in a microbiological oven for 24 h (overnight) at the temperature of 37°C^14^^,^ 16. At the end of the growth time, the BHI broth solution containing the bacteria was placed in a centrifuge for 10 min at 4°C and 9,500 rpm. Then, the supernatant was discarded, and the pellet formed was resuspended in 5 mL of sterile phosphate buffered saline.

Serial dilution of this bacterial solution was performed to count the colony forming units (CFUs) per gram of tissue. Aliquots were prepared in cryotubes (2-mL capacity) containing 800 μL of the *Pseudomonas* broth with 3.4 × 109 CFU·mL^-1^ and 200 μL of sterile 80% glycerol. These aliquots were immediately stored in a -20°C-freezer and used in all further experiments in this study.

### Biofilm induction

Biofilm induction on the wounds was performed by applying a sterile cellulose filter (membrane) with a thickness and porosity of 0.2 μm, measuring 5 mm in diameter, in which 10 μL of the bacterial suspension containing 10^4^ CFU·mL^-1^ was applied. The control group received the same filter with 10 μL of phosphate buffered saline (sterile vehicle) to mimic and simulate wound infection.

### Sample

The animals were provided by the Vivarium Center (CEBIO) of UFMG. To carry out the experimental procedures, the animal welfare parameters were considered according to the recommendations of Technical Guidance No. 12, from the year 2018, of the National Council for the Control of Animal Experimentation of Brazil.

The mice were healthy, isogenic, male, C57BL/6 mice, aged approximately 8 to 12 weeks and with a body weight between 20 and 30 g. The sample size was calculated from the analysis of a 95% confidence interval for the mean (significance level of 5%) and an 80% chance of detecting a difference between the means, that is, a statistical power of the test of 80%, considering a *n* sample of eight animals for each experimental group. A 20% error and replacement percentage of sample loss over the *n* were considered.

The animals were randomly divided into two study groups, called control group (Pbs) and intervention or infected group (Pa). The proposed procedures were applied to both groups, and time follow-up occurred at five different times: 0, three, five, seven and 10 days, totaling a *n* sample size of 64 animals.

### Surgical and anesthetic procedure

The animals were anesthetized and then submitted to hair trichotomy with a minitrichotomizer (Wall, portable model) and asepsis of the skin on the dorsal region with 70% alcohol solution. Subsequently, four wounds were made on the mid-back region of these mice with the aid of a sterile surgical punch with a diameter of 5 mm^15^ to remove the entire extent of the skin tissue, including epidermis, dermis, hypodermis, and panniculus carnosus. The wounds were made with a minimum distance of 0.5 cm and a maximum of 1 cm between them in both horizontal and vertical positions^17^.

### Data collection

The experimental procedures were carried out from January 2019 to July 2020.

In the animals of the control group, 10-μL phosphate buffered saline (vehicle) was applied, while the animals of the infected group received 10 μL of the bacterial suspension containing 10^4^ UFC·mL^-1^. In both groups, the respective solutions were applied to the cellulose filters placed over the four wounds, as adapted from the biofilm infected wound model^14^. The filters were attached to the wounds with a strip of transparent polyurethane film measuring 3 cm long by 15 cm wide to maintain contact with the wound bed during the first 24 h of the experiment. The polyurethane film was applied to cover all four wounds in the dorsal region to allow the entire dorsoventral region of the animals to be involved^17^.

At the end of the first 24 h, the filters were removed from the wounds, and the wounds were again occluded with the transparent film. This cover was changed on days 3, 5, and 7 of each experimental time period to measure and evaluate the wounds, and then the transparent film was reapplied. The end of the experimental follow-up occurred on the tenth day.

### Measurements

Day 0 (day 0, baseline) was when the wounds were made, and the other times were considered as Day 3 (3-day follow-up), Day 5 (5-day follow-up), Day 7 (7-day follow-up), and Day 10 (10-day follow-up) after the wounds were made. These experimental times are important markers for monitoring biofilm induction and formation in the wound healing process.

The wound closure profile was performed by photographing and measuring the area of the wounds on days 0, 3, 5, 7 and 10. A digital caliper (Insize) was used to measure the wound area. The results were expressed as percentage of closure relative to the original wound size, using the Eq. 1 15:


100−(current wound area) / (original wound area)×100 (1)

The counterevidence of the presence of *P. aeruginosa* in the wound bed was performed by means of the macerate culture technique of the tissues corresponding to the wound area on days 0, 3, 5, 7, and 10. Skin corresponding to the wound area was weighed and normalized to 100 mg of tissue per 2,000 μL (2 mL) of phosphate buffered saline. The wound macerate was diluted in this solution using the serial dilution technique at a ratio of 1:1,000. Then, 100 μL of this solution was plated onto Petri dishes containing cetrimide agar, a microorganism culture medium selective for the isolation of *P. aeruginosa* in biological samples of animal origin^18^^,^ 19. Subsequently, these plates were incubated for 24 h at 37°C to ensure amplification and growth of the CFUs. Results were expressed as mean ± standard error of the mean representing the number of CFU·g^-1^ of tissue.

For the evaluation of clinical signs of sepsis in the animals, the techniques of liver tissue macerate culture were used at three, five and seven days and total and differential leukocyte counts at days 0, 3, 5, 7 and 10 after the induction of local infection in the wounds. Liver tissue was weighed and normalized to 100 mg of tissue to 1,000 μL (1 mL) of phosphate buffered saline. The techniques for maceration, dilution, and culture of liver tissue were applied identically to the procedures described before for wound culture. The white blood cell count was evaluated by collecting blood from the mouse’s tail, with 5 μL of blood being considered for the total white blood cell count and 5 μL for the smear on a glass slide for the differential cell count. The results obtained (CFU·g^-1^ of tissue and number of leukocytes per mL of blood) were expressed as mean ± standard error of the mean.

Clinical follow-up of the animals was performed by daily clinical inspection, measurement of body weight described in grams on days 0, 3, 5, 7 and 10 before the anesthetic procedure and macroscopic evaluation of the wounds on days 0, 3, 5, 7 and 10 considering the presence of odor, coloration of necrotic tissue, presence of shiny, translucent and viscous film on the surface of the wounds^7^^-^ 9, presence of loose and fluid necrotic tissue (sphacel) or presence of devitalized tissue of gelatinous appearance^10^^,^ 11.

The biofilm formation profile and identification of the biofilm on the wounds were evaluated by inspecting the wounds under a transmission electron microscope (TEM) (Tecnai G2-12 model, FEI SpiritBiotwin 120 kV).

### Statistical analysis

The survival curve of the animals was determined using the Kaplan–Meier method to estimate the probability of survival at various time intervals. The log-rank test (Mantel–Cox) was used to compare survival curves between both groups.

For calculation purposes, the mean area ± standard error of the mean of the wounds at all experimental times was used.

Statistical analyses were performed in GraphPad Prism software version 6.0, and results are represented as mean ± standard error of the mean. Values of p < 0.05 were considered significant. For comparisons between two groups over time, two-way analysis of variance (ANOVA) was used, followed by Tukey’s multiple comparisons test with the purpose of checking the interaction between the dependent variables over time. For comparisons between three or more groups, one-way ANOVA was used, followed by Tukey’s multiple comparisons test in order to check the interaction between the independent variables over time.

## Results

**Figure 1:**
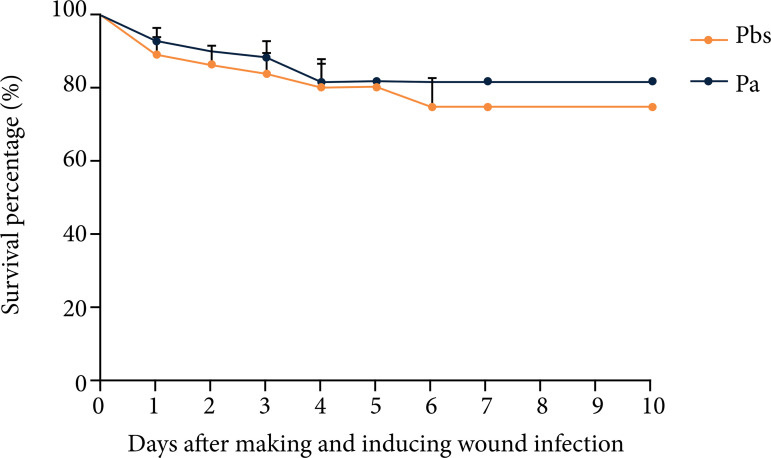
Mice survival curve after wound dressing and infection (2019)*.

The evaluation of animal survival after wound dressing and induction of infection detected no statistically significant difference between the two tested groups (Fig. 1).

*P: 0.5742 (log-rank, Mantel–Cox test); Pbs: control group; Pa: intervention or infected group.

There were no statistical differences in the percentage of survival between the two groups evaluated (p = 0.5742). There were animal losses in both groups, totaling eigth deaths in the Pbs group (*n*_i_ = 37 animals) and seven deaths in the Pa group (*n*_i_ = 42 animals) throughout the experimental follow-up. These numbers, when compared to the *n* initial (*n*_i_) applied for each group, equate to a mortality percentage of 21.62% in the Pbs group and 16.67% in the Pa group.

**Figure 2:**
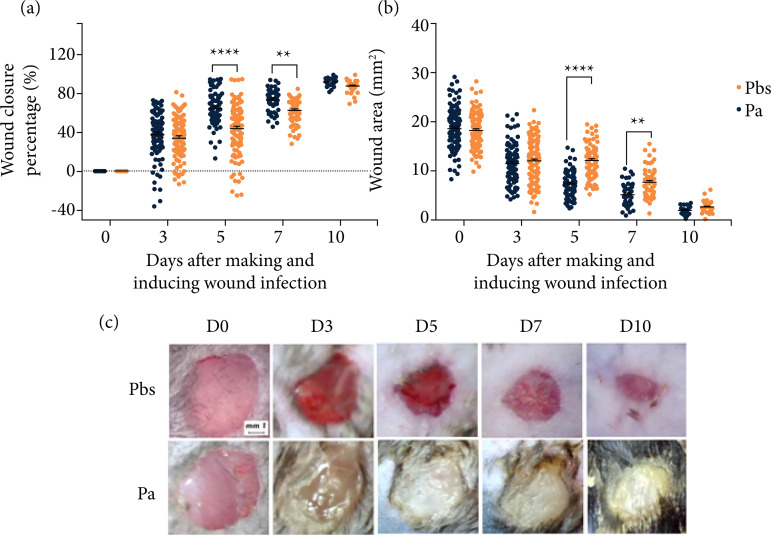
Wound closure kinetics and area assessment (2020): **(a)** percentage of wound closure; **(b)** wound area; **(c)** wound at each assessment time^#^.

The wound closure profile demonstrated that the wounds in the Pa group showed lower wound healing percentage and respectively larger wound area when compared to the Pbs group with a statistically significant difference at D5 and D7 after wound preparation and induction of wound infection (Fig. 2).

Pbs: control group; Pa: intervention or infected group; ^#^Pbs *vs*. Pa, results are described as mean ± scanning electron microscope; **p < 0.01; ****p < 0.0001.

The mean percentage of wound closure in the Pa group was 43.40% (± 2.61 scanning electron microscope–SEM) at D5 and 62.48% (± 1.67 SEM) at D7. In the Pbs group, this percentage reached 64.64% (± 1.75 SEM) on D5 and 73.96% (± 1.51 SEM) on D7. In the wound area analysis, it was observed that the Pa group had a mean wound area of 12.13 mm^2^ (± 0.39 SEM) on D5 and 7.67 mm^2^ (± 0.35 SEM) on D7. The Pbs group had a mean area of 7.36 mm^2^ (± 0.29 SEM) and 5.01 mm^2^ (± 0.29 SEM) on D5 and D7, respectively.

**Figure 3:**
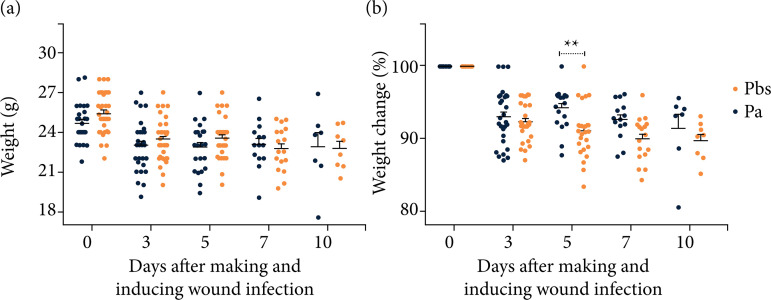
Evaluation of weight and ponderal variation (2019-2020): **(a)** gross weight of animals; **(b)** ponderal variation of animals^#^.

The clinical-ponderal follow-up detected no statistically significant differences between the Pbs and Pa groups regarding the animals’ gross weight (Fig. 3a). However, more pronounced weight variation was observed until D3 in both groups throughout the experimental follow-up. From D3 on, the weight variation between groups occurred less intensely, and there was a statistically significant difference at D5 (p < 0.01) when compared to D0. The difference between the average percentage of weight loss at this time point was 9% (± 0.71 SEM) in the Pa group and 6% (± 0.69 SEM) in the Pbs group (Fig. 3b).

Pbs: control group; Pa: intervention or infected group; ^#^Pbs *vs*. Pa assessments; **p < 0.01.

**Figure 4:**
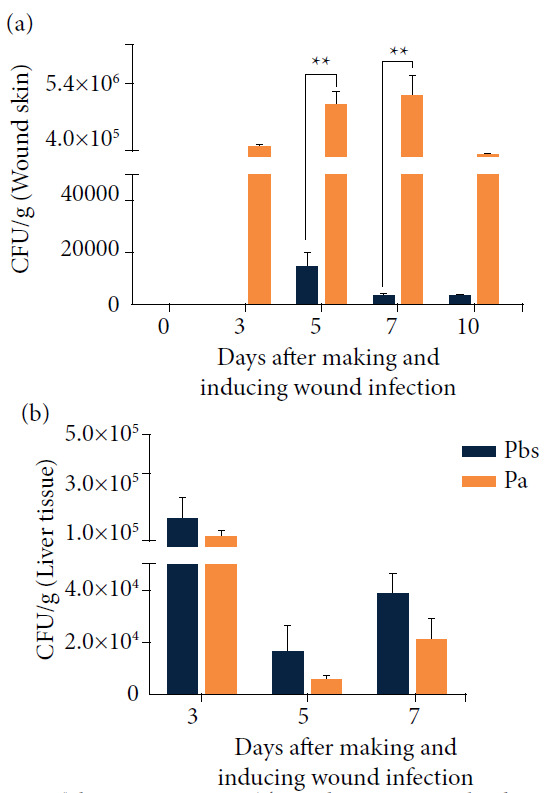
Wound and liver tissue culture (2019-2020): **(a)** skin culture corresponding to the wound area; **(b)** hepatic tissue culture^#^.

The skin plating and culture techniques employed allowed the recovery of large amounts of *P. aeruginosa* (CFU·g^-1^) in the tissue corresponding to the wound area at D3, D5, D7 and D10 and a low amount of these microorganisms in the Pbs group at D5, D7 and D10 (Fig. 4a), with statistically significant differences at D5 and D7 in the comparison between the two groups analyzed (p < 0.01).

In the evaluation of liver tissue culture, no differences with statistical significance were observed between the two groups evaluated (Fig. 4b).

Pbs: control group; Pa: intervention or infected group; ^#^Pbs *vs*. Pa assessments. The results were expressed as the mean ± scanning electron microscope**p < 0.01.

**Figura 5:**
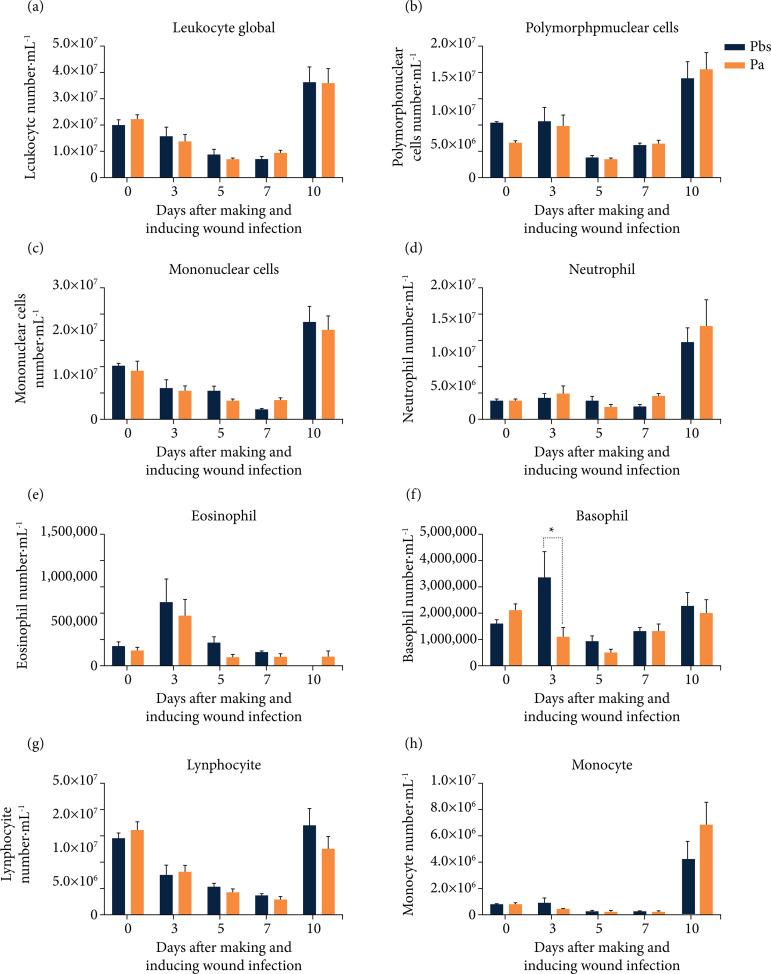
Global and differential leukocyte quantification in peripheral blood (2019-2020): **(a)** global leukocyte assessment; **(b)** polymorphonuclear cell assessment; **(c)** mononuclear cells; (**d, e, f, g, h**) differential quantification of leukocytes: neutrophils, eosinophils, basophils, lymphocytes, and monocytes, respectively^#^.

Analysis of the overall quantification of leukocytes between the Pbs and Pa groups showed no statistically significant differences (Fig. 5a). The same situation was observed in the quantification of polymorphonuclear leukocytes (Fig. 5b) and mononuclear (Fig. 5c). In these three analyses, a lower number of cells were observed at the time of D5 and D7, followed by an exponential increase in these cells at D10 of experimental follow-up when compared to baseline cellularity levels (D0) in both groups.

There were no statistical differences in the quantification of neutrophils and eosinophils. A peak of neutrophils was observed on D10 (Fig. 5d), while for eosinophils this peak occurred on D3 after surgical trauma and induction of infection (Fig. 5e). In both cases, there was reduction in the number of these cells at D5 and D7. Basophil quantification was statistically significant at D3 with p < 0.05 between the two groups evaluated (Pbs *vs*. Pa). There was also a marked reduction in the number of these cells at D5 in both groups studied with a return to levels similar to the baseline level (D0) at D10 (Fig. 5f).

It was observed that the lymphocytes showed significant reduction in the number of circulating cells on D3, D5 and D7 when compared to D0. However, on D10 of the experiment, these cells return to values close to baseline in both groups evaluated (Fig. 5g). As well, a peak of monocytes was noticed on the tenth day after wounding and induction of infection (Fig. 5h), without statistical significance in both cases.

Pbs: control group; Pa: intervention or infected group; ^#^Pbs *vs*. Pa in the graph. The results were expressed as the mean ± scanning electron microscope; *p < 0.05.

**Figura 6:**
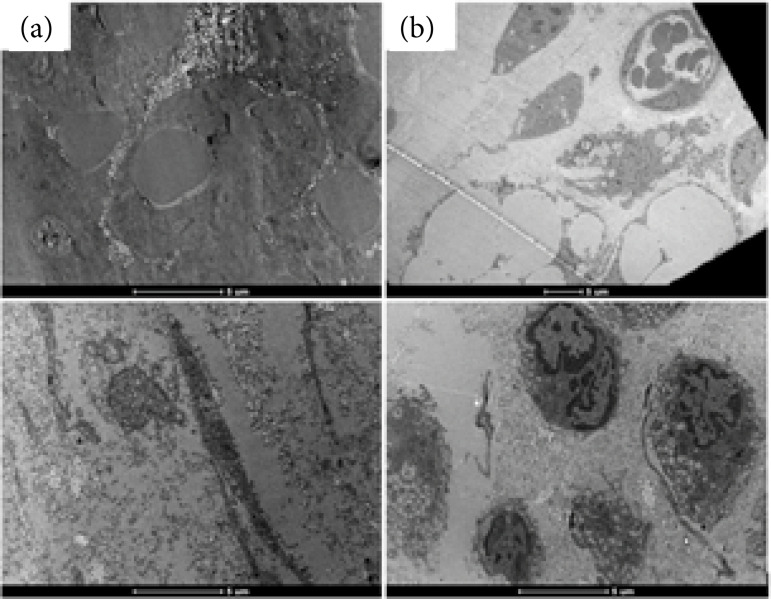
Representative images of the absence of biofilms in the wound area (2020): **(a)** Pbs (control group); **(b)** Pa (infected with *P. aeruginosa* group)*.

In the evaluation of the wounds using transmission electron microscopy (TEM), it was not possible to identify the presence of bacterial biofilm or *P. aeruginosa* in its planktonic form (Fig. 6).

Pbs: control group; Pa: intervention or infected group; *in both groups no biofilm or bacterial cells were observed in the wound area. Scale bar: 5 μm.

**Figura 7:**
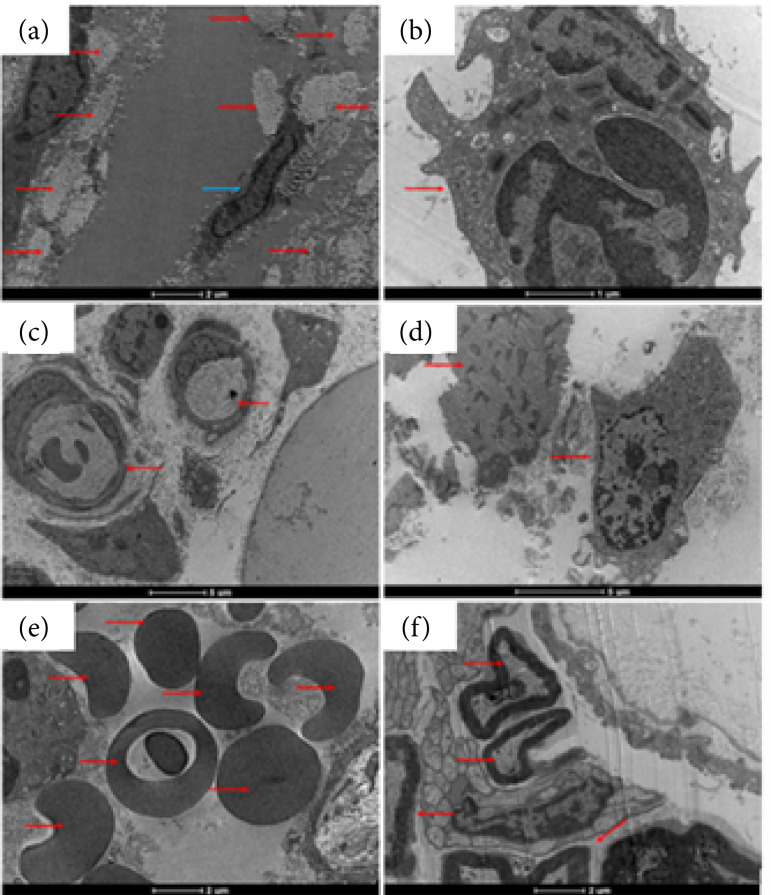
Representative images of the absence of biofilms in the wound area (2020): **(a)** Pbs (control group); **(b)** Pa (infected with *P. aeruginosa* group)*.

Some characteristic and well-defined cellular structures were identified in the tissue corresponding to the wound area, such as the presence of collagen fibers, immune system cells, the presence of nerves, blood vessels, and cells in the process of apoptosis (Fig. 7).

Pbs: control group; Pa: intervention or infected group; *scale bar: at (A, E, F): 2 μm; at (B): 1 μm; at (C, D): 5 μm.

**Figura 8:**
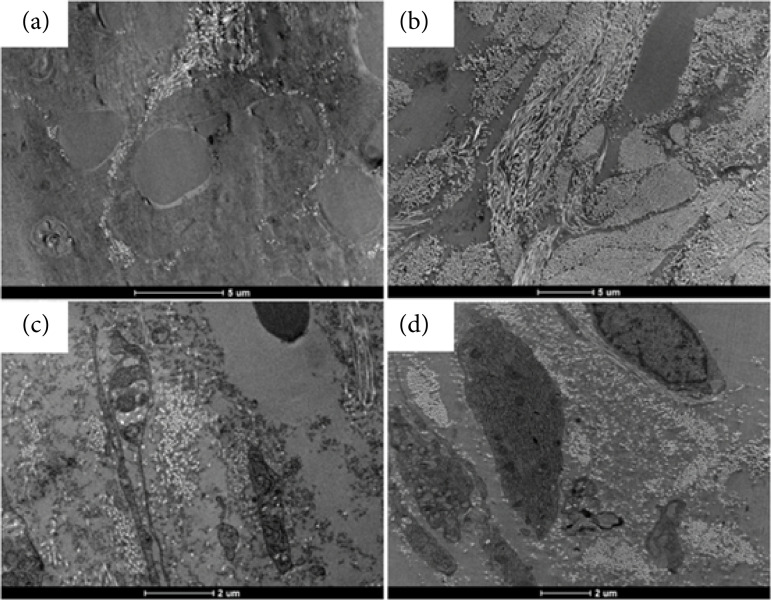
Images of the wounds by transmission electron microscopy: collagen fiber deposition in the wound area. **(a)** Pbs (control group) to a 5 μm scale; **(b)** Pa (infected group) to a 5 μm scale; **(c)** Pbs to a 2 μm scale; **(d)** Pa to a 5 μm scale.

Among the observed characteristics, it is noteworthy that the deposition of collagen fibers was more prominent in the Pa group when compared to the Pbs group (Fig. 8).

Pbs: control group; Pa: intervention or infected group; *collagen is visualized as bright, white dots scattered in the tissue.

**Figura 9:**
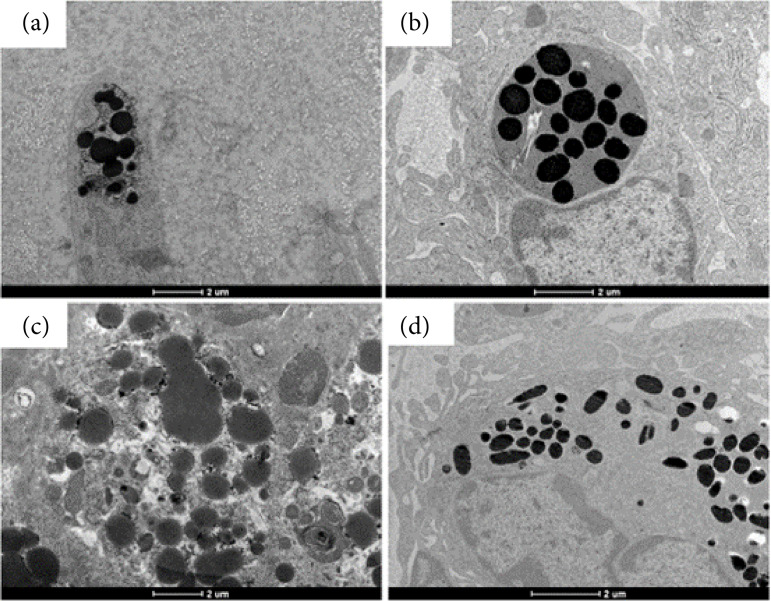
Images of the wounds by transmission electron microscopy: cells with electrodense granules (2020); (**a and c**) Pbs (control group), cells containing electrodense granules; (**b and d**) Pa (infected group), cells containing electrodense granules*.

TEM allowed us to identify different cells such as the presence of fibroblasts, eosinophils and neutrophils containing electrodense granules dispersed in the cytoplasm and vacuolized cells with well-defined electrodense granules within the structure of the vacuoles in both groups evaluated, suggestive of substances synthesized by the cells as a response to the inflammation process arising from the surgical process and induction of infection with *P. aeruginosa* in the wound region (Fig. 9).

*Scale bar: 2 μm.

## Discussion

When searching for other similar models in the literature, a wide variety of proposals were identified, each with different variables that could impact the results, altering the expected outcomes. These findings allowed us to rethink some strategies that led to the standardization of a model of wounds colonized or infected by biofilms that was viable and reproducible, allowing from the identification of the biofilm to the most different research situations around this theme.

The wound model was associated with different bacterial loads already described in the literature by other authors working on this same topic^14^^,^ 20 ^-^ 23. However, the bacterial load of 10^4^ CFU·mL^-1^ was selected for induction of infection and biofilm formation.

The evaluation of the wound samples by TEM did not allow the identification of bacterial biofilm. No bacteria were identified in their planktonic form. The same methodology was applied in a study by other authors^14^ and allowed the presence of biofilm to be identified only in the crust region of the wounds in proportions almost 100 times higher than the result observed in wound culture, unlike the study^21^ in which the presence of *P. aeruginosa* was detected entangled in an extracellular matrix on the surface of the porcine wound (burn) model by SEM.

Other researchers have devoted themselves to the induction and formation of the biofilm in murine^24^. In this study, an infection load and an infection induction technique were described in a manner quite similar to the techniques employed in this research. The positive result of the wound culture (CFU·mL^-1^) was considered as the proof that biofilm was formed in the wound bed. In the publication, there is no information that allows the presence of biofilm and how its structural identification was performed^24^.

The balance between collagen synthesis and degradation occurs around four weeks after the injury, initiating the maturation phase of these fibers, characterized by the replacement of type III collagen by type I collagen, which can last for months or even years^25^^,^ 26.

The presence of cells such as fibroblasts, eosinophils, and neutrophils with scattered electrodense granules in the cytoplasm and cells with vacuoles containing these granules well delimited within the structure of the vacuoles were suggestive findings that the samples evaluated were in the inflammatory and proliferative phase of the healing process^25^. The presence of these granules was more prominent in the Pa group when compared to the Pbs group.

The most studied microorganisms associated with wound infection are *Staphylococcus aureus* and *P. aeruginosa* 27. These data support the choice of the excisional skin wound model performed in this investigation and support the choice of *P. aeruginosa* as a biofilm inducing source.

Despite the limitations of *in-vitro* and *in-vivo* nonmammalian surrogate models, they continue to be widely used, revealing important information about the physiology and treatment of biofilms^27^. However, there is a strong questioning in the scientific community about the multiplicity of models of infected wounds described in recent years because of the irreproducibility of these models in everyday research^28^^,^ 29. The difficulty of reproducing the model was noted in the research’s initial stage due to the high mortality of animals contaminated with the bacterial load cited in the reference study^30^.

Publications present a growing body of evidence suggesting that bacterial biofilms represent an important mainstay in the pathogenesis of chronic wounds^31^. They further signal that the main barriers to running *in-vitro* research models include the failures related to reproducing the host environment and the ethical issues surrounding the use of animals in *in-vivo* 27 research as opposed to a small amount of clinical research.

In the present study, the delay in the wound healing process in the Pa group at five and seven days also coincided with the increased bacterial load observed for this same period in the wound culture evaluation. This is different from what occurred in the study evaluating the percentage of epithelialization of partial thickness porcine wounds^20^, in which there were no statistical differences between the control and infected groups at the times evaluated. These authors report a slight tendency toward a higher epithelialization rate on D3, but without statistical significance.

Increased bacterial load is considered one of the causes of chronicity of skin lesions, especially when biofilm formation occurs^8^^,^ 9 ^,^ 11 ^,^ 24 ^,^ 32 ^,^ 33. In clinical practice, the delay in the wound healing process with critical colonization or recurrent infection is also perceived in patients with wounds of various etiologies^34^, a situation that can be aggravated by the inefficient eradication of infectious and opportunistic pathogens^35^, such as bacterial biofilms.

Researchers have devoted themselves to discussing the microbiota of wounds, according to the classification into chronic^32^^,^ 36 ^,^ 37 and acute wounds^36^^,^ 38. Among the etiologies, there are venous ulcers^36^^,^ 39, pressure injuries^36^^,^ 39 and diabetic foot ulcers^36^^,^ 37 ^,^ 39. These authors support the hypothesis that bacterial populations present in the wound bed are strongly associated with the etiology of lesions^32^^,^ 36 ^,^ 40 and, consequently, with biofilm formation.

The choice of *P. aeruginosa* to induce infection in the wound model adopted in this research is based on the fact that it is one of the main biofilm-forming bacterial microorganisms. From this perspective, some authors point out that the virulence of *P. aeruginosa* is controlled by a highly complex signaling network involving everything from natural resistance to antimicrobials, to the expression of extracellular appendages^41^.

The virulence of biofilms is associated with the type of microorganism involved in their formation^37^. The main mechanisms include acting as a reservoir of pathogenic cells for inoculation into the bloodstream, the release of excessive amounts of endotoxins by the cells that compose them, and tissue damage caused by the excessive reaction of immune system components to the biofilm, in addition to the provision by the biofilm of an ecological niche for the evolution of antibiotic resistant organisms^42^.

In this sense, for the clinical evaluation of biofilm in wounds, some authors advocate the characteristic presence on the wound surface: shiny, translucent, and viscous film^7^^-^ 9, loose sphacel, of gelatinous consistency that quickly rebuilds after removal^8^^,^ 10 ^,^ 11. These criteria were considered in the present study as an auxiliary tool in the evaluation of the macroscopic characteristics of the wounds. However, this assessment alone is not able to determine the location or even the presence of biofilm in the wounds.

Regarding the variation in body weight of the animals over the days, a significant difference was observed between the Pbs and Pa groups at the 5-day time point. Such a fact allows to infer that anesthetic-surgical trauma in conjunction with high bacterial load may interfere with the healing process and weight recovery, contributing to wound chronicity. A finding corroborated by another study, whose authors^43^ related increased wound area and marked weight loss when wounds were inoculated with a high bacterial load immediately after their confection, using the biofilm transfer infection induction model. However, it was identified that the point of the infection induction model of this study was less aggressive, with animals experiencing a lower mortality rate when compared to the biofilm transfer model employed by these authors.

These data support the hypothesis that patients with chronic wound infection may develop persistent weight loss, since there is increased metabolism and reports of inappetence. However, this hypothesis is not clear and requires further studies to be proven. As a way to implement research in this regard, it is suggested that the systematic evaluation of weight variation be included in the anamnesis and care processes of patients with chronic wounds, considering that these components are directly related to the healing process. The findings can be associated with the most requested laboratory tests during the control and clinical follow-up of patients in the health services.

The data from the global and differential quantification of leukocytes indicate that in the acute wounds of healthy mice, even considering the bacterial load that was applied to the wounds, they are able to debulk the inflammatory process, preventing the onset of chronicity. However, this result is not confirmed for the wound assessment of patients seen in the various health care services. The chronicity of these wounds, especially venous ulcers, is directly related to the persistence or exacerbation of the inflammatory phase^44^. Excess bacterial load has been considered one of the main justifications for the chronicity state of wounds or skin ulcers^45^.

Mature bacterial biofilms are capable of modulating host immune response by reducing or inhibiting phagocytosis by immune system cells, antimicrobial resistance; production and secretion of toxins capable of promoting a persistent hypoxic and inflammatory microenvironment in the wound^11^^,^ 46 ^-^ 48. Some of these characteristics of mature biofilms were observed in this study by the marked reduction of leukocytes (neutrophils, eosinophils, basophils, lymphocytes, and monocytes) on D5 and D7, as well as by the increased bacterial load and worsening (stagnation) of the healing process in this same period. Such conditions raised the following questions: was the bacterial load employed in the study capable of forming biofilm? Is the biofilm formed modulating the immune response as described in the literature? Is the observed reduction in monocytes related to reduced phagocytosis in biofilm infected wounds? However, a detailed investigation of these questions was not feasible at the current stage of the research and will be investigated in the next stages of this study.

When searching for answers in the literature to explain the findings of this study, one came across the scarcity of publications covering the standardization of wound models with biofilm. Such a gap may delay the construction of robust knowledge on the subject. Another limitation observed in the evaluation of the *in-vivo* studies in the animal model relates to the lack of discussion in the literature surrounding the mortality rate of animals after wound infection with pathogenic microorganisms.

This study allowed us to establish the infection induction technique and the safe bacterial load to induce local wound infection while keeping the animal safe. However, it was not possible to prove the presence of biofilm by means of TEM, making the model limited for product evaluation in biofilm removal. The lack of confirmation of the presence of biofilm in the wound urges the researchers to continue the study for further adjustments.

The divergences between the findings of the studies reinforce the need to review the preparation and processing technique of the wound/skin samples for TEM or require the association of other ancillary techniques for the ultrastructural identification of biofilms. The TEM evaluation of the samples allowed us to identify some characteristics in the cellular structures corresponding to the wound area, for example, the presence of collagen fibers, immune system cells, the presence of nerves, blood vessels, and cells in the process of apoptosis. Among these observed characteristics, the presence of collagen fibers and cells containing electrodense granules stood out.

Reflecting on the results obtained in this study, we have as expectations for the next phase of the research the hypothesis of evaluating the application of a reinforcement of the bacterial load to ensure the maintenance of infection and/or colonization of the wounds by biofilm for a longer period of time, in order to implement the testing of products used in the treatment of infected wounds with biofilm. This condition is necessary, considering that the adopted wound model, regardless of the bacterial load applied, leads to wound closure between the 10th and 14th day after surgical trauma.

In this sense, the research intends to review the standard fixation technique employed in the preparation of skin samples in this phase of the study, aiming to search for some biomarker that facilitates the identification of biofilm microcolonies in the wound, as well as admits the possibility of associating other sample preparation techniques such as cryofixation and rapid freezing, immunohistochemistry techniques and fluorescence microscopy, for example, that may favor the demarcation of the specific wound site to be evaluated by TEM.

## Conclusions

The results obtained confirm that the proposed infected wound model with bacterial load of 10^4^ CFU·mL^-1^ was able to slow down the healing process, simulating critical colonization in excisional wounds of mice, as well as being safe to ensure the survival of animals throughout the experimental design.

The point infection induction model was less aggressive compared to the biofilm transfer model employed in other studies.

The recovery of *P. aeruginosa* in the wound tissue at D5 and D7, when associated with the slowing of the healing process in the animals in the Pa is a strong indication of colonization of the wounds by *P. aeruginosa.* However, the evaluation by means of TEM did not detect the presence of biofilm or *Pseudomonas* in its planktonic form in the investigated wounds. The findings raise questions about the understanding of the ease of formation and the high occurrence of biofilm in chronic wounds.

The results confirm the need for continuation of this study for adjustments in the biofilm infected wound model and improvement of its detection techniques.
